# An Argument in Favor of Deep Brain Stimulation for Uncommon Movement Disorders: The Case for N-of-1 Trials in Holmes Tremor

**DOI:** 10.3389/fnhum.2022.921523

**Published:** 2022-06-17

**Authors:** Marcelo Mendonça, Gonçalo Cotovio, Raquel Barbosa, Miguel Grunho, Albino J. Oliveira-Maia

**Affiliations:** ^1^Champalimaud Research and Clinical Centre, Champalimaud Foundation, Lisbon, Portugal; ^2^NOVA Medical School, Universidade Nova de Lisboa, Lisbon, Portugal; ^3^Department of Psychiatry and Mental Health, Centro Hospitalar de Lisboa Ocidental, Lisbon, Portugal; ^4^Department of Neurology, Centro Hospitalar de Lisboa Ocidental, Lisbon, Portugal; ^5^Department of Neurology, Hospital Garcia de Orta, Almada, Portugal

**Keywords:** Holmes tremor, deep brain stimulation, connectivity, movement disorders, n-of-1 trials

## Abstract

Deep brain stimulation (DBS) is part of state-of-the-art treatment for medically refractory Parkinson’s disease, essential tremor or primary dystonia. However, there are multiple movement disorders that present after a static brain lesion and that are frequently refractory to medical treatment. Using Holmes tremor (HT) as an example, we discuss the effectiveness of currently available treatments and, performing simulations using a Markov Chain approach, propose that DBS with iterative parameter optimization is expected to be more effective than an approach based on sequential trials of pharmacological agents. Since, in DBS studies for HT, the thalamus is a frequently chosen target, using data from previous studies of lesion connectivity mapping in HT, we compared the connectivity of thalamic and non-thalamic targets with a proxy of the HT network, and found a significantly higher connectivity of thalamic DBS targets in HT. The understanding of brain networks provided by analysis of functional connectivity may thus provide an informed framework for proper surgical targeting of individual patients. Based on these findings, we argue that there is an ethical imperative to at least consider surgical options in patients with uncommon movement disorders, while simultaneously providing consistent information regarding the expected effectiveness and risks, even in a scenario of surgical-risk aversion. An approach based on n-of-1 DBS trials may ultimately significantly improve outcomes while informing on optimal therapeutic targets and parameter settings for HT and other disabling and rare movement disorders.

## Introduction

Deep Brain Stimulation (DBS) is commonly used in the treatment of movement disorders (Krack et al., 2019). Specifically, it has a very well-defined role in the management of motor symptoms in patients with medically refractory Parkinson’s Disease and Essential Tremor (Krack et al., 2019). The data supporting patient selection criteria and therapeutic indication comes from a vast number of clinical trials and longitudinal observational studies performed in these common and relatively well characterized movement disorders (Krack et al., 2019). Data supports a population effect, although there is high inter-individual variability in response to treatment (Santaniello et al., 2018). Dystonia is another major indication for DBS (Fox and Alterman, 2015). It is a disease characterized by abnormal muscle contractions and posturing, and is less frequent, and possibly more heterogeneous, than PD and ET. Trials have revealed effectiveness of DBS to treat dystonia (Vidailhet et al., 2005), with longitudinal studies revealing the sustainability of long-term effects, and clear superiority when compared to medical therapy. In the United States, DBS for dystonia is approved by the Food & Drug Administration (FDA) under a humanitarian Device Exemption (Miocinovic et al., 2013). However, RCT-based DBS effectiveness is mostly clear for generalized, segmental or focal dystonia, with earlier onset and a probable genetic cause (Vidailhet et al., 2005; Miocinovic et al., 2013; Fox and Alterman, 2015).

Other causes of parkinsonism, tremor and dystonia exist, and they may develop not only in the context of degenerative disease but also after a static brain lesion. Following a severe head injury, 12.2% of the surviving patients develop a movement disorder (Krauss et al., 1996), most commonly tremor and/or dystonia. Also after stroke, 3.7% of patients develop a movement disorder in the first year (Alarcón et al., 2004), most commonly tremor, dystonia or chorea. These movement disorders usually differ on lesion locations but share a common general clinical phenomenology. Holmes tremor (HT) is a classic example of these post-lesional syndromes. It is a debilitating condition, characterized by a rest and intention tremor, with a relatively large amplitude and a slow-frequency (less than 4.5 Hz) (Raina et al., 2016). This tremor may also have a postural component and have a delayed onset after an insult to the Central Nervous System (CNS). Treatment of HT is frequently unsatisfactory, resulting in a major burden for patients (Krauss et al., 1996; Alarcón et al., 2004; Krauss, 2015).

In this article we use HT to illustrate that, based on the risk-effectiveness profile, there is an imperative to consider DBS for medically-refractory movement disorders developing after lesions of the central nervous system. Specifically, we propose that N-of-1 trial designs could overcome the limitation of randomized controlled trials to assess the effectiveness and risks of DBS in these rare conditions among heterogeneous patient populations, and performed a simulation to support this proposal. Finally, preliminary analyses of the current neuroscientific understanding of brain networks underlying HT were conducted to test if this could provide an informed framework for proper targeting of individual patients.

## The Limitations on State-Of-Art Medical Treatment of Holmes Tremor

HT is a condition usually presenting either to general neurologists or movement disorders specialists. HT is responsive to levodopa in around 50% of cases (Raina et al., 2016; Wang et al., 2020) with Raina et al. (2016) reporting a near complete control in 7/24 patients (30%) treated with levodopa. In the remaining patients, anecdotal responses were found to topiramate, levetiracetam, trihexyphenidyl, phenobarbital, amantadine, clonazepam, bromocriptine or quetiapine (Raina et al., 2016; Wang et al., 2020). Tentative therapeutic approaches have included pramipexole, lamotrigine, flunarizine, carbamazepine, propranolol, baclofen, gabapentin, valproic acid and piracetam (Striano et al., 2007; Raina et al., 2016; Rojas et al., 2018; Wang et al., 2020). Unlike levodopa, therapeutic effects are not seen with any of these drugs, and in general, responses are considered poor or absent. However, anecdotal reports of response to a specific drug frequently lead clinicians into off-label use of different agents at high dose, alone or in combination. Minimally invasive medical approaches, such as botulinum toxin, have also been tested, but only with mild-to-moderate effectiveness (Latino et al., 2015; Kreisler et al., 2019). Thus, if levodopa fails there is no single effective agent to be tried, leading clinicians to a strategy of sequential drug trials, in a trial-and-error approach performed across long periods of time in each patient.

**FIGURE 1:**
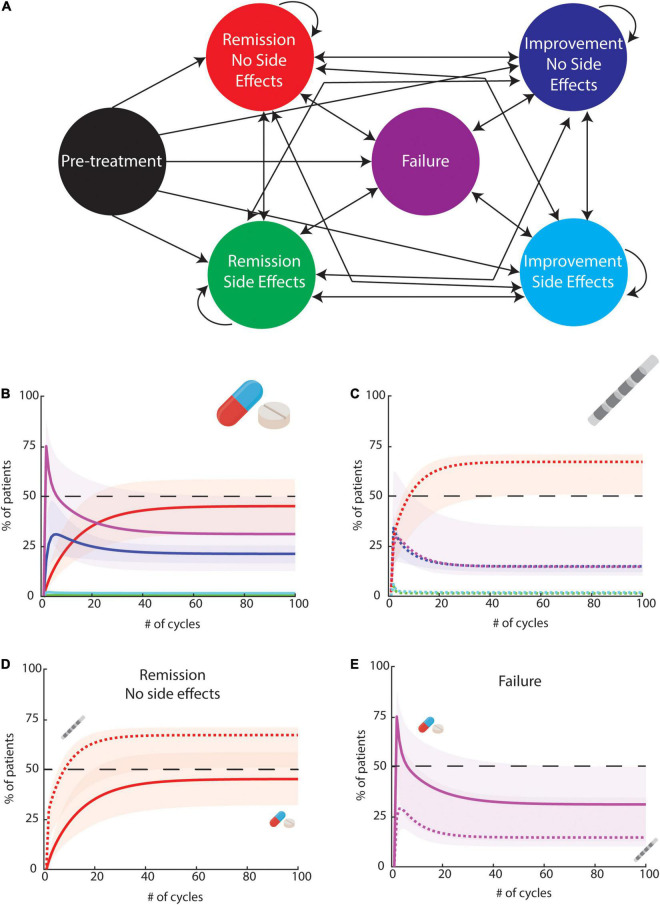
A six-state Markov Model to study the effects of multiple medical treatment trials or DBS trials followed by therapeutic optimization. **(A)** State definitions: each circle represent a state. Arrows represent possible transitions. Transition probabilities are described in Supplementary Tables 1, 2. The first state is never visited after the first cycle, so it was excluded from the panels bellow. **(B)** Result of the simulation of 100 cycles for medical therapy. The full lines represent the main model using the transition probabilities described in Supplementary Table 1. Shaded color includes the interval of the results between 2 additional models using the same assumptions as the main model but with effectiveness reduced by a factor or 2, or increased by a factor of 2 **(C)** Result of the simulation of 100 cycles for DBS. The dashed lines represent the main model using the transition probabilities described in Supplementary Table 2. Shaded color includes the interval of the results between 2 additional models using the same assumptions as the main model but with effectiveness reduced by a factor or 2 or increase by 20% (further increases were considered unrealistic based on available data). **(D)** Result for remission without side effects for medical therapy (full line) and DBS (dashed line). We found that 66.5% of DBS responses were superior to the range of medical responses. **(E)** Result for failure without side effects for medical therapy (full line) and DBS (dashed line). 40.5% of possible medical responses were found to be worse than DBS responses.

To understand the general effectiveness of this medical therapy trial-and-error approach after levodopa failure, we performed a simulation using a Markov Chain analysis (Figure 1) of sequential single agent trials. A Markov chain is a model that describes a sequence of possible events (state-transitions) in which the probability of the event depends only on the previously reached state. This model assumes that, at a specific time, a patient is always in one out of a finite number of discrete health states, and it can be used to model sequences of decisions. Markov models have the potential to inform real-world decisions that more faithfully represent clinical problems than, for instance, decision trees (Sonnenberg and Beck, 1993). We designed a six states model and assumed that after a significant symptom remission without side effects there was a very high probability of maintaining that state (96%). We estimated known efficacy from previous reports (Striano et al., 2007; Raina et al., 2016; Rojas et al., 2018; Wang et al., 2020) and for medical therapy, we considered a 5% rate of significant symptomatic remission (R), 20% of improvement (I) and 10% of side effects (SE). Two additional models – one with 2 times higher effectiveness, and one with 2 times lower effectiveness were also performed, to account for potential under or overestimation. We defined the 6 states, namely pre-intervention state, remission with SE, remission without SE, improvement with SE, improvement without SE, and treatment failure (both with and without SE; please see details in Figures 1A,B and transition probabilities in Supplementary Table 1).

Our simulations suggest that 17 tentative trials (cycles) would be necessary to lead to a significant symptomatic remission in over 33% of patients, and seven cycles needed to lead to any improvement in the absence of SE in at least 50% of the subjects. Considering that a proper trial (including dosage titration and time to assess symptoms remission) would imply at least four months of follow-up, we can estimate that we would need more than five years to promote symptomatic remission at least 33% of patients. In the first two years, we do not expect to be able to significantly improve more than 47% of patients using this strategy (with less than 15% achieving remission). It is important to highlight that these numbers are optimistic estimates as they assume that one out of four patients experience any kind of improvement. Also, it is unlikely that the 17 different agents have the same probability of effectiveness. Importantly, these optimistic estimates are disappointing for both physicians and patients, that must deal with low therapeutic effectiveness, and choices of drugs not clearly based on pathophysiological mechanisms, leading to uninformed trial-and-error strategies.

## The Role of DBS in the Treatment of Holmes Tremor

While motor symptoms are thought to emerge from dysfunction of certain brain circuits, other than the specific response to levodopa, unsurprising due to the description of lesions in the Substantia Nigra pars compacta dopaminergic pathway (Seidel et al., 2009), there is no clear unifying theme, including receptor or circuit-specific actions, across the other agents (Remy et al., 1995; Gajos et al., 2017). On the other hand, linking DBS with brain network dysfunctions is more plausible. In fact, small case series and isolated reports have found that DBS may have a role in the treatment of uncommon movement disorders, namely tremor after brain lesions (Mendonça et al., 2018; Wang et al., 2020). A recent review on DBS for lesional tremor described a median improvement of 75% in tremor scores across 82 cases (Mendonça et al., 2018). Considering the 52 cases where individualized patient data was provided, 11 had a near to complete response to treatment (defined as improvement above 90%). After Levodopa, DBS may thus be a generalizable and effective approach in treating HT. Another recent systematic review found a significantly higher improvement for DBS-treated HT patients in comparison with medically treated ones (including levodopa; respectively, 7.35 ± 2.01 vs. 6.06 ± 2.58 points on a 10-point scale, *p* = 0.025) (Wang et al., 2020).

To compare hypothetical effectiveness of medical options and DBS we expanded our Simulation approach (Figure 1A) creating an additional simulation for DBS. We kept the assumption that after a significant symptom remission without SE there was a very high probability of maintaining that state (96%). We then considered a 35% rate of significant symptomatic remission (R) (vs. 5% in medical arm), 40% of improvement (I) (vs. 20% in medical) and 15% of side effects (10% in medical - details in Figure 1C and Supplementary Table 2). Data was estimated from previous work (Mendonça et al., 2018; Wang et al., 2020). As previously done, two additional models – one with 20% higher effectiveness, and one with 2 times lower effectiveness were also performed. While we needed 17 cycles to have over 33% of subjects achieving a significant remission (irrespective of presence of SE) with medical therapy, one cycle would be enough in the DBS arm - reducing the predicted five years time to promote symptomatic remission in at least 33% of patients to just one step with the surgical procedure. In this scenario, the number of patients effectively treated is expected to be improved with trials of stimulation parameters changes. Regarding remission without side effects across the multiple models, 66.5% of DBS responses were superior to the medical range (Figure 1D). Regarding failure rates, 40.5% of possible medical responses were worse than DBS responses (Figure 1E). Importantly, in the absence of controlled trials, these data-driven simulations provide support for the putative effectiveness of DBS in HT. While available data was insufficient for additional simulations, we nevertheless also would like to mention availability of lesion-based therapies as ablative surgery with MRI-guided Focused Ultrasound. Thalamotomy by MRgFUS has been explored as a therapy for treatment refractory essential tremor (Abe et al., 2021) and other atypical tremor disorders (Fasano et al., 2017) with descriptions of significant and sustained tremor relief.

## Tools for an Informed Framework of DBS in HT

While the simulations described above are supportive of the use of DBS for HT, the technique remains challenging. As stated above, there is high variability in the lesion locations, which has probably led to heterogeneity in DBS target selection (Mendonça et al., 2018; Wang et al., 2020). In fact, the mechanisms behind DBS are not fully characterized. Although there is evidence supporting that it is very likely that stimulation induces action potential generation (or disruption) in axons near the electrode, there is still lack of understanding on the general network effect and how network changes affect movement and treat dysfunction of movement (Gradinaru et al., 2009; Chiken and Nambu, 2016). However, novel techniques addressing brain connectivity have provided novel insights into key surgical targets. A recent functional connectivity mapping study showed that brain lesions leading to HT were connected to a common brain circuit with nodes in the red nucleus, thalamus, globus pallidus pars interna and cerebellum (Joutsa et al., 2019). This network was specific for HT and distinct from other lesions causing non-HT movement disorders. The authors described that, in seven HT patients submitted to a focal neurosurgical procedure, the target was functionally connected with the network emerged from causal brain lesions.

When DBS is used for treatment of HT, the thalamus has been a privileged target, comprising 57.8% (Wang et al., 2020) to 63.6% (Gajos et al., 2017) of the total cases reported. Thalamic targets have included the Ventral Intermedius nucleus (VIM), the Ventral oral posterior nucleus (VOP) and Ventral oral anterior nucleus (VOA). We reviewed the literature and identified 7 publications (Samadani et al., 2003; Goto and Yamada, 2004; Oyama et al., 2011; Issar et al., 2013; Kobayashi et al., 2014; Kilbane et al., 2015; Boccard et al., 2016) where DBS was used to treat HT that provided detailed information on electrode coordinates. In these 7 publications, a total of 18 electrode locations were reported (9 thalamic, 6 GPi and 3 STN targets). For one case the coordinates were reported in the MNI space. In the remaining cases, conversion to MNI space coordinates was performed according to previous publications (Eickhoff et al., 2009). Each of these coordinates was considered as the center of regions of interest for further analysis, each consisting of 3 mm radius spheres centered on the respective coordinates, and thus simulating Volumes of Tissue Activated (VTAs), as performed previously (Cotovio et al., 2020) and according to available literature (Maks et al., 2009). Then, as performed in the context of lesion network mapping (Fox, 2018; Cotovio et al., 2020), for each simulated VTAs, using resting state functional MRI data from the Human Connectome Project (Glasser et al., 2013) (*N* = 937), we computed correlations between the average activity of each VTA location and the activity of every brain voxel. To obtain the final network map for each VTA, we averaged the results across all individuals of the connectome and Fischer z transformed the correlation maps. Finally, we obtained the connectivity of each VTA to the HT connectivity map reported by Joutsa et al. (2019) by summing the intersection of each VTA network map with the HT connectivity map spatial component, which was computed by creating 3 mm spheres centered on each one of the Centers of Gravity (CoG), as reported by Joutsa et al. (2019).

**FIGURE 2:**
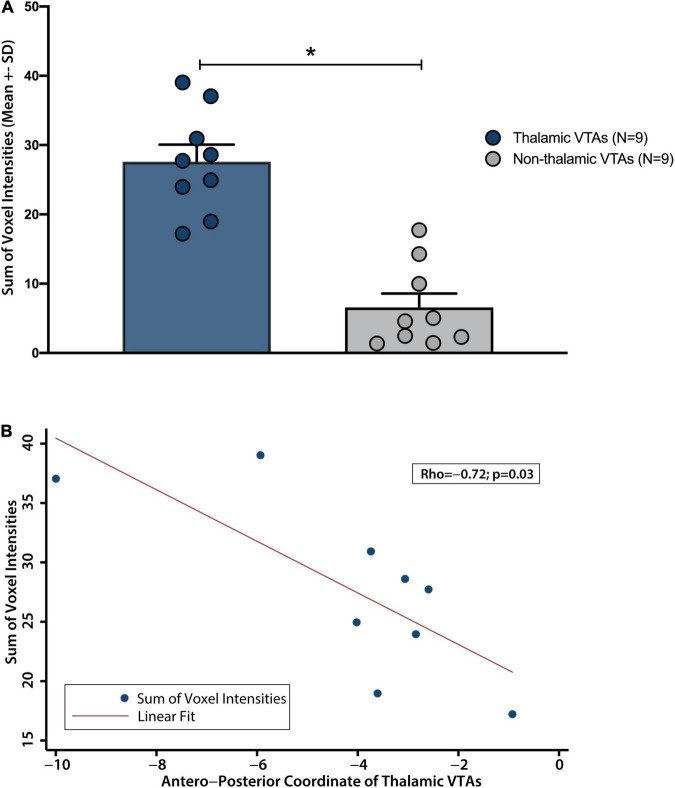
Connectivity of VTAs with the spatial component of the previously descibed HT lesion network map. **(A)** Thalamic VTAs have a significantly higher connectivity with the spatial component of previously described HT lesion network map than non-thalamic VTAs. **(B)** Connectivity varied significantly along thalamic antero-posterior axis. *p < 0.001.

Using this approach, we found that thalamic VTAs had higher connectivity with a network formed by the HT CoGs than non-thalamic VTAs (Z = 3.488, p < 0.001, Wilcoxon rank-sum test; Figure 2A). Interestingly, in thalamic VTAs, we also found high variability in connectivity even when the contact locations are very similar, with three-dimensional cartesian standard deviation of thalamic coordinates as low as 2.50mm. While these analyses support the use of thalamic targets for DBS in HT, they also support that, at least in the case of the thalamus, different contacts on the same DBS electrodes, where centers of adjacent electrode contacts are spaced between 2 and 3 mm, may modulate activity in distinct brain networks. In line with this, the connectivity of thalamic VTAs to the HT CoG network was significantly correlated with the antero-posterior electrode position (Figure 2B, rho = −0.72, *p* = 0.03). While preliminary, our findings support that, as multiple patients undergo DBS for HT worldwide, a n-of-1 trial approach may be not only feasible but a desirable approach, allowing for optimization of DBS in HT patients, as described further below.

## The Relevance of a N-of-1 Trial Approach

Everyday clinical practice usually involves “therapeutic trials” for individual patients. These informal trials of treatments are part of usual care, unblinded, and have no control conditions (Duan et al., 2013). They are susceptible to bias and uncertainty in their assessment (Gabler et al., 2011). N-of-1 trials overcome these vulnerabilities, at least partially. They are prospectively planned multiple crossover trials conducted in a single individual, of particular use when symptoms are stable and treatment takes effect quickly with minimal carryover effects. N-of-1 trials utilize multiple comparisons with a control condition and *a priori* decision about the outcome choice and assessment timing (Gabler et al., 2011; Duan et al., 2013; Margolis and Giuliano, 2019). Most of these conditions are found in DBS in HT. After a surgery (with a single electrode implant) patients may go through multiple trials assessing stimulation location (using one or more of the 4-to-8 contacts typically available, or the benefits of directional electrodes), stimulation frequency, pulse width and quantity of current delivered.

Since electrode positioning is a key variable for successful DBS, *a priori* information about relevant circuits can contribute to planning and/or interpretation of N-of-1 trials. Individual therapeutic responses to different contacts/stimulation parameters may be assessed and compared to individual connectivity maps of the specific lesion causing the HT in that patient (Cole et al., 2022). Furthermore, evidence from multiple individual effective DBS contacts could ultimately be compared to the one generated by lesion mapping analysis, as done in preliminary fashion above. Systematically assessing the effects of individualized responses to different electrode positions (and different volumes of tissue activated - VTA) would create datasets that could then be summarized across patients to inform the treatment of HT. This approach may inform and further expand on a recently proposed approach where a symptom network target library is established based on group-level data, and the circuits to target could be matched to individual patient circuits (Hollunder et al., 2022). This may also build a robust database that can guide stimulation parameters optimization. N-of-1 trials are particularly well placed to inform clinicians on target and stimulation parameters selection. Although we still cannot infer causality from these approaches, they could eventually, and powerfully, inform target selection. Such approaches would additionally allow exploring optimal symptomatic/phenotypical responses for each patient and, finally, this theoretical framework can possibly be generalized to other movement disorders.

## Discussion

People are usually risk-averse for loss (Kahneman and Tversky, 1979). When choices are placed between a pharmacological agent and a surgical procedure, patients may express risk aversion toward surgery (Cykert, 2004). This is particularly evident for patients with a non-life threatening condition, such as movement disorders, that regard surgery as a possible cause of major losses in autonomy (Kim and Jeon, 2019) or even death. A similar phenomenon may be happening with physicians, that tend to overestimate surgical risks (Healy et al., 2018), and that may bias surgical risk assessment (Ferguson et al., 2019). It is therefore necessary to have proper data to assess DBS risks and benefits, in comparison to those of non-surgical approaches. In a series of 728 patients with multiple diagnosis operated by a single surgeon between 2002 and 2010, major SE leading to hemiparesis and/or decreased consciousness occurred in 13 patients (Fenoy and Simpson, 2014), leading to the proposal that overall risk of procedure- and hardware-related adverse events is acceptably low. Previous data also suggests that DBS may be very effective for HT, and our simulation suggests an effectiveness that is higher than an approach of sequential pharmacological agents. This needs to be discussed with levodopa-refractory patients, with need of an effective therapeutic option for their tremor as evidence on new targets emerge (Fox and Deuschl, 2022).

However, clinicians should keep in mind that DBS for HT still involves an optimal patient and target selection. HT is a very heterogeneous condition, frequently paired with other neurological symptoms, namely ataxia, known to have a relatively poor response to DBS. Management of the proximal and intention tremor components in HT is challenging, not only due to the potentially associated ataxia but also due to a limited effect of thalamic stimulation in proximal musculature (Wang et al., 2020). Although some improvement in tremor may happen in these patients, it may have a negligible impact on their general motor function. It is also possible that, even if a common connectivity node is shared across HT patients, the impact of heterogeneity of lesions across multiple circuits could affect therapeutic response/side-effect profile for individual patients. Structural changes in the brain caused by lesions may also perturb the surgical targeting. Individual patient outcomes should thus not be reduced to modeling, and even considering our models, failure is still expected in approximately 1 in 5 HT patients submitted to DBS. Finally, it is important to underline that this simulation approach was not designed to identify who are the patients that are not expected to improve. Despite these limitations, a paired and iterative approach based on n-of-1 trials of DBS and connectivity mapping of responses could enlighten therapies for HT and other highly disabling and uncommon movement disorders. Connectivity based approaches have been similarly explored in psychiatric disorders (Cotovio et al., 2020; Cole et al., 2022), and can provide a design more feasible than that of a randomized controlled trial.

## Data Availability Statement

The raw data supporting the conclusions of this article will be made available by the authors, without undue reservation.

## Author Contributions

MM, GC, and AJO-M conceived and designed the work. MM was responsible for conducting the Markov model simulations and was responsible extracting coordinates from the literature. GC was responsible for conducting the connectivity analysis. MM, GC, RB, and AJO-M analyzed and interpreted the data. MM drafted the manuscript with GC and AJO-M and it was critically revised by the remaining authors for important intellectual content. All authors contributed to the article and approved the submitted version.

## Conflict of Interest

AJO-M was national coordinator for Portugal of a non-interventional study (EDMS-ERI-143085581, 4.0) to characterize a Treatment-Resistant Depression Cohort in Europe, sponsored by Janssen-Cilag, Ltd. (2019–2020), is recipient of a grant from Schuhfried GmbH for norming and validation of cognitive tests, and is national coordinator for Portugal of trials of psilocybin therapy for treatment-resistant depression, sponsored by Compass Pathways, Ltd. (EudraCT number 2017-003288-36 and 2020-001348-25), and of esketamine for treatment-resistant depression, sponsored by Janssen-Cilag, Ltd. (EudraCT NUMBER: 2019-002992-33). The remaining authors declare that the research was conducted in the absence of any commercial or financial relationships that could be construed as a potential conflict of interest.

## Publisher’s Note

All claims expressed in this article are solely those of the authors and do not necessarily represent those of their affiliated organizations, or those of the publisher, the editors and the reviewers. Any product that may be evaluated in this article, or claim that may be made by its manufacturer, is not guaranteed or endorsed by the publisher.
