# Supplementary material for: An Argument in Favor of Deep Brain Stimulation for Uncommon Movement Disorders: The Case for N-of-1 Trials in Holmes Tremor
Source: Front Hum Neurosci. 2022 Jun 17;16:921523. doi: 10.3389/fnhum.2022.921523 (PMC9247189; doi:10.3389/fnhum.2022.921523)
Supplement: Supplementary file 1 [file Data_Sheet_1.docx]

| **To**  **From** | **Start** | **R – No SE** | **R – SE** | **I – No SE** | **I – SE** | **Failure** |
| --- | --- | --- | --- | --- | --- | --- |
| **Start** | 0 | 4,5 | 0,5 | 18 | 2 | 75 |
| **R – No SE** | 0 | 96 | 1 | 2 | 1 | 0 |
| **R – SE** | 0 | 7,2 | 20,8 | 14,4 | 1,6 | 56 |
| **I – No SE** | 0 | 1,35 | 0,15 | 67,2 | 0,8 | 30,5 |
| **I – SE** | 0 | 3,6 | 0,4 | 21,6 | 22,4 | 52 |
| **Failure** | 0 | 4,5 | 0,5 | 18 | 2 | 75 |

**Supplementary Table 1 -** Transition probabilities across the 6 states for medical therapy arm. This assumes a probability of 5% for remission, 20% for improvement and 10% of side effects. If the state of remission without side effect was reached, the probability of maintaining this state would be of 96%. If remission was reached but with side effect, we assumed a 20% bias of keeping this state, and an additional 10% probability of remission (10% of the remaining 80%: 8%; these 8% are distributed with 10% for the presence of side effects - an additional 0,8% - and 90% with the absence of side effects - 7.2%). If improvement was reached but with side effect, we assumed a 20% bias of keeping this state, and an additional 30% probability of improvement (30% of the remaining 80%: 24%; these 24% are distributed with 10% for the presence of side effects - an additional 2.4% - and 90% with the absence of side effects - 21.6%). These higher probabilities were considered due to the fact that a positive response (even when paired with side effects) could inform subsequent agent choices (for higher effectiveness agents). If the patient improved in the absence of side effects, we considered a 60% bias to maintain this state.

| **To**  **From** | **Start** | **R – No SE** | **R – SE** | **I – No SE** | **I – SE** | **Failure** |
| --- | --- | --- | --- | --- | --- | --- |
| **Start** | 0 | 29,75 | 5,25 | 34 | 6 | 25 |
| **R – No SE** | 0 | 96 | 1 | 2 | 1 | 0 |
| **R – SE** | 0 | 20,4 | 23,6 | 23,8 | 4,2 | 28 |
| **I – No SE** | 0 | 6,8 | 1,2 | 70,2 | 1,8 | 20 |
| **I – SE** | 0 | 6,8 | 1,2 | 13,6 | 22,4 | 56 |
| **Failure** | 0 | 8,5 | 1,5 | 17 | 3 | 70 |

**Supplementary Table 2 -** Transition probabilities across the 6 states for DBS. For the first cycle (surgery moment) probabilities were set to 35% for remission, 40% for improvement and 15% of side effects. After this first step we considered the same biases as before (20% for Remission with side effects and Improvement with side effects and 60% for improvement without side effects) but reduced the probabilities of remission and improvement according to the state. In the remission (with Side Effects) state, probability of remission decreased to 30% and improvement to 35%. In the Improvement without side effects state, probability of remission was reduced to 20% and improvement to 30%. In the improvement with side effects and failure states, probabilities of remission were reduced to 10% and improvement to 20%.
